# Segregationally stabilised plasmids improve production of commodity chemicals in glucose-limited continuous fermentation

**DOI:** 10.1186/s12934-022-01958-3

**Published:** 2022-11-03

**Authors:** James R. Allen, Mario A. Torres-Acosta, Naresh Mohan, Gary J. Lye, John M. Ward

**Affiliations:** 1 Advanced Centre for Biochemical Engineering, University College London, Gower Street, London, WC1E 6BT UK; 2 Tecnologico de Monterrey, School of Engineering and Science, Av. Eugenio Garza Sada 2501 Sur, C.P. 64849 Monterrey, N.L México

**Keywords:** Stabilised plasmids, Continuous culture, Cer sequence, Bio-based chemical production

## Abstract

**Background:**

The production of chemicals via bio-based routes is held back by limited easy-to-use stabilisation systems. A wide range of plasmid stabilisation mechanisms can be found in the literature, however, how these mechanisms effect genetic stability and how host strains still revert to non-productive variants is poorly understood at the single-cell level. This phenomenon can generate difficulties in production-scale bioreactors as different populations of productive and non-productive cells can arise. To understand how to prevent non-productive strains from arising, it is vital to understand strain behaviour at a single-cell level.

The persistence of genes located on plasmid vectors is dependent on numerous factors but can be broadly separated into structural stability and segregational stability. While structural stability refers to the capability of a cell to resist genetic mutations that bring about a loss of gene function in a production pathway, segregational stability refers to the capability of a cell to correctly distribute plasmids into daughter cells to maintain copy number. A lack of segregational stability can rapidly generate plasmid-free variants during replication, which compromises productivity.

**Results:**

Citramalate synthase expression was linked in an operon to the expression of a fluorescent reporter to enable rapid screening of the retention of a model chemical synthesis pathway in a continuous fermentation of *E. coli*. Cells without additional plasmid stabilisation started to lose productivity immediately after entering the continuous phase. Inclusion of a multimer resolution site, cer, enabled a steady-state production period of 58 h before a drop in productivity was detected. Single-cell fluorescence measurements showed that plasmid-free variants arose rapidly without cer stabilisation and that this was likely due to unequal distribution of plasmid into daughter cells during cell division. The addition of cer increased total chemical yield by more than 50%.

**Conclusions:**

This study shows the potential remains high for plasmids to be used as pathway vectors in industrial bio-based chemicals production, providing they are correctly stabilised. We demonstrate the need for accessible bacterial ‘toolkits’ to enable rapid production of known, stabilised bacterial production strains to enable continuous fermentation at scale for the chemicals industry.

**Supplementary Information:**

The online version contains supplementary material available at 10.1186/s12934-022-01958-3.

## Introduction

Production of commodity chemicals at industrial-scale relies on chemical synthesis using fossil fuels as precursors [22]. This is a highly efficient and productive process, refined over many years of incremental innovations in the chemicals industry. However, price fluctuations in the fossil fuel market and the increased global scrutiny on their use in large industrial process are creating strong drivers towards bio-based production and sustainable ‘green’ processes [6, 17]. The shift to bio-based processes is hindered by the lack of true continuous processes (i.e., continuous fermentation) [7]. Batch fermentation involves steps with lengthy reactor downtime during set-up, sterilisation and cleaning. These non-productive periods result in lost potential revenue and have additional costs, which ultimately limit profitability sufficiently to make these processes economically unfeasible. A continuous process has limited reactor downtime, providing productivity can be retained for sufficiently long periods. In order to retain this productivity, new techniques, strains and genetic tools are required that can be used directly by the chemicals industry, for any particular product of interest [7]. Extended production timescales for continuous fermentation are reliant on strains containing stable pathway genes, with consistent expression levels.

Methacrylic acid is a chemical with various uses as a precursor to polymers widely used in plastics. It demonstrates a growing market of 8% per year with economics that suggest biological production could be feasible [21]. Citramalate is a potential precursor to methacrylic acid via a hot pressurised water decarboxylation/dehydration and previous experiments have demonstrated production of citramalate in *E. coli* by the single addition of the CimA3.7 enzyme [33].

The stability of a plasmid in a carbon-limited fermentation, without selective pressure is largely governed by plasmid loss at division (segregational stability), with, on average, approximately 3% of a population becoming plasmid free in each generation [5]. Stabilisation of a pathway by genomic insertion is another method that has been widely studied [8, 16, 30, 32]. It typically results in a lower gene dosage number, which can hinder product yields and extends R&D timescales by requiring insertion and subsequent quality control on every prototype pathway proposed. The addition of an antibiotic selective pressure to plasmid-borne pathways in an industrial-scale reactor vessel is prohibitively expensive and a significant hazard in the event of accidental release. In industrial-scale continuous bacterial systems, sufficient reaction length can only be obtained by ensuring sufficient gene dosage is maintained, which in turn, requires stabilising the plasmid without external additives.

**Fig. 1:**
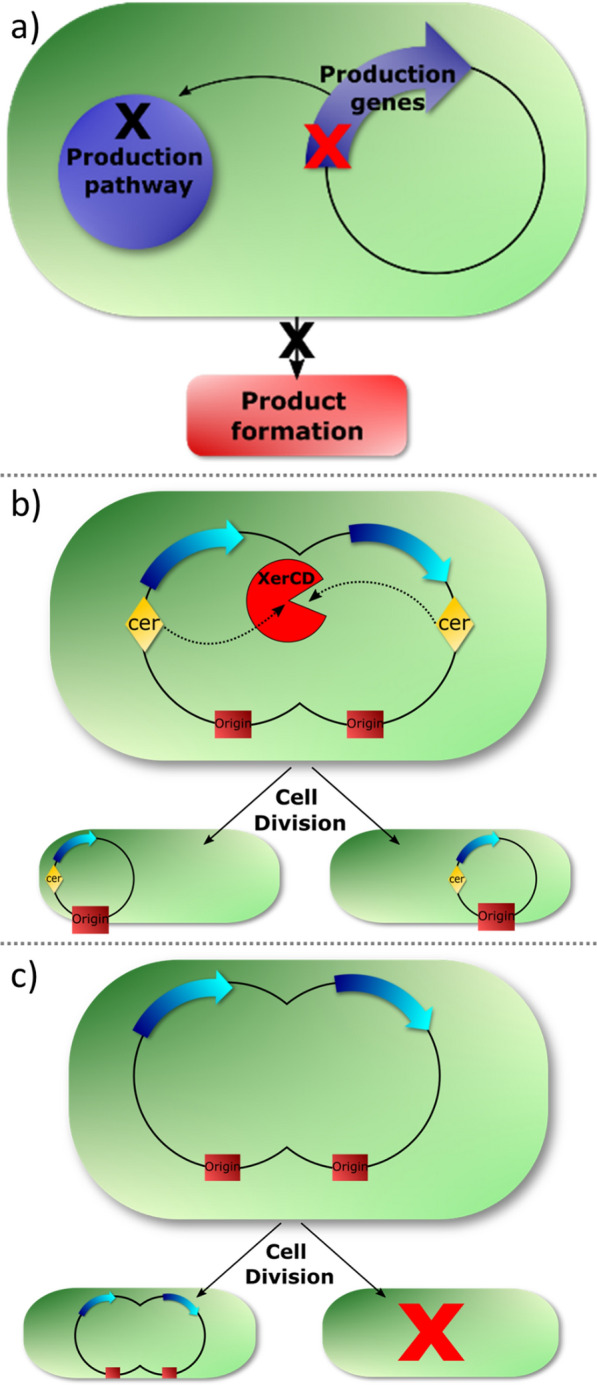
**a** A structural mutation in the production genes (red cross) causes non-function in the production pathway resulting in no product formation from the affected cell. **b** Plasmid dimer formation during replication results in uneven plasmid distribution in daughter cells and a subpopulation that no longer contains the plasmid harbouring production genes (red cross) and therefore no longer forms product. **c** The XerCD system of *E. coli* separates plasmid dimers, limiting the possibility of daughter cells becoming plasmid-free

The persistence of genes located on plasmid vectors is dependent on numerous factors, but can be broadly separated into structural stability and segregational stability [9, 12, 34]. Structural stability is lost with genetic rearrangements or mutations that cause a loss of vector or operon function (Fig. 1a). Segregational stability is lost when vectors are segregated incorrectly into the daughter cells (Fig. 1b). Structural instability results in sub-populations capable of using more of the available carbon for growth, as genes for chemicals production are lost or inactivated. Segregational instability results in decreased numbers of cells in a population containing the plasmid, which also lowers the growth constraints for subsequent generations as gene dosage and thus metabolic burden of a production pathway are lost in the plasmid free cells.

Previous studies have shown the cer fragment to have an effect on plasmid retention for industrial production [11]. cer is a target site for the XerCD system in *E. coli*, which is a multimer resolution system, enabling daughter plasmids to properly segregate by recombining across the duplicated cer sites in a plasmid dimer to generate monomers [29]. During repeated growth phases, significant plasmid-free cells arise within 24 h. The addition of cer mitigates this loss. Other stabilisation systems seek to kill cells where the plasmid has not segregated into, they don’t replace the missing part of the plasmid’s replication and segregation machinery removed early in the genealogy of the vector. In determining the effects of cer integration, cell counts from selective and non-selective agar plates do not determine how cer achieves this mitigation, nor do they show how hosts containing a plasmid harbouring a cer site still eventually lose productivity.

The advent of synthetic biology has allowed rapid generation of gene vectors and allows combinatorial studies that can generate greater breadth of coverage [18]. Type IIS restriction assemblies are a good example of this. They allow combinatorial assemblies of DNA ‘parts’ in a consistent order with high levels of efficiency [1, 10, 24, 27, 28]. Here we use a Type IIS restriction endonuclease-based system to develop plasmids with a model industrial process (citramalate production gene, cimA) and a fluorescent reporter both under constitutive expression. This allowed the linking of the pathway gene to a reporter for tracking of plasmid retention by placing both genes in an operon.

Single-cell analysis of plasmid retention can give a vastly greater understanding of the populations arising during an industrial fermentation process. Traditional plasmid retention assays have relied on detecting the retention of selectivity markers, such as antibiotics, on colonies resulting from agar plate growth. Flow cytometry is a potential powerful analytical tool that allows the detection of fluorescence in individual particles [23]. In this case, it means that expression of the production-linked Red Fluorescent Protein gene can be determined at a single cell level. This allows the presence of actively expressing plasmid to be determined and compared between different strains. It does not require an additional growth step (which does occur when counting colonies) and it can also analyse fluorescence expression levels. The latter gives a greater depth of information about whether plasmid is still present and whether it is still productive.

The use of single-cell analysis techniques here, allowed the direct comparison of plasmid retention with and without a cer fragement. This fragment provided an exemplary method for plasmid stabilisation for long-term, continuous fermentations. Single-cell fluorescence expression analyses were compared to citramalate production to determine the effectiveness of the fluorescence marker as a proxy for monitoring industrial production pathway gene retention. As a result, maintenance of production was tracked throughout a 106 h continuous fermentation at 1-L scale. Retention of fluorescence and citramalate production were shown to increase with addition of cer stabilisation, resulting in total yield increases of over 150%. Here we demonstrate that cer stabilisation can be applicable to an industrially-relevant continuous fermentation, by ensuring mis-segregation is not a factor in resultant plasmid loss. We show that single-cell analyses are vital to understanding precise mechanisms of plasmid plasticity in cells, enabling a rational understanding of follow-up steps to further-stabilise plasmids towards timeframes that maximise productivity for industrial bio-based commodity chemical production.

## Results

### One-pot assembly of a model reporter chemicals production plasmid and a stabilised variant

**Fig. 2:**
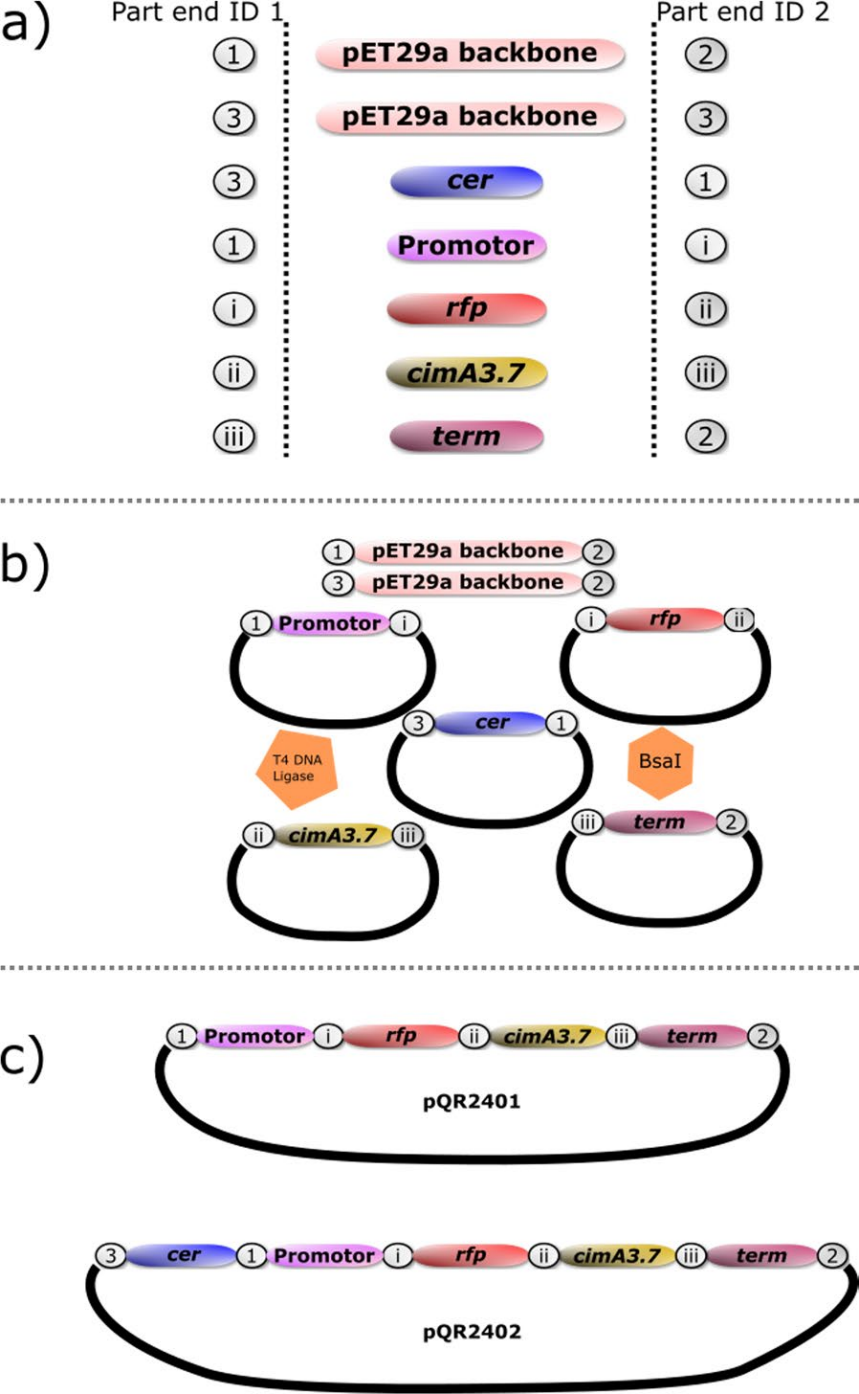
The layout of the genes and the Type IIS part ends used to make up plasmids pQR2401 and pQR2402, ends fully explained in Additional file 1. **A** The different parts used in the assembly aligned with their respective ‘part ends’ to create complementary overhangs for the correct assembly order. The backbone of pET29a was amplified with either ‘Part End 1’ (for pQR2401) or ‘Part End 3’ (for pQR2402) at one end and always ‘Part End 2’ at the other. **B** The resulting components used in a TypeIIS digestion/ligation one-pot assembly. The pentagon indicates the presence of T4 DNA ligase and the hexagon indicates the presence of the TypeIIS restriction Endonuclease BsaI. The ‘Parts’ described in (**a**), with the exception of the backbone, were all ligated into a storage vector prior to this step, as indicated by the solid line representing a circular DNA molecule. **c** The only complete plasmids that can arise from the described one-pot assembly. pQR2401 assembles with a promotor followed by the *rfp* and *cimA3.7* genes and a terminator region (shortened to ‘term’). pQR2402 assembles with the same operon arrangement but with a *cer* multimer resolution region upstream

Type IIS restriction assembly techniques were used to assemble two variant plasmids for citramalate expression and reporting. This allowed the exact order of the operon components to be arranged specifically and also assembled all in a ‘one-pot’ process. Figure. 2 shows the components and products of this assembly. A pET29a( + ) backbone was chosen as a widely used expression cassette, from which a 3573 bp sized fragment of the backbone containing the rop and kanamycin resistance (kanr) genes, as well as the pBR322 origin, was cloned by PCR and purified by gel electrophoresis. The fresnoRFP gene was assembled into a plasmid alongside the cimA3.7 gene to generate a reporter linked to a model chemicals production pathway, both of which were expressed under the Anderson collection constitutive promotor BBa_J23119 [19]. This generated the plasmid pQR2401, described in Fig. 2. The cer fragment was included as a further DNA part in the assembly upstream of the production-reporter operon to generate plasmid pQR2402, as described in Fig. 2. This arrangement allows for any combination of pathway genes and/or reporters to be assembled using the same method, generating a potential tool for the easy generation of industrially relevant strains in the future.

### Retention of expression throughout continuous fermentation

BW25113 ΔldhA cells were transformed with either of the resultant plasmids pQR2401 or pQR2402, the same host strain described previously as demonstrating high levels of product formation [33]. Cells were then grown in a glucose-limited chemostat for 106 h, in defined media, without selective pressure to determine the length of production of the chemical citramalate, determined by HPLC.

Glucose concentration in the chemostat dropped for the first 12 h until steady state was reached, and the reactor became carbon-limited. Glucose levels then remained constant until the end of the fermentation. Citramalate production from cells containing plasmid pQR2401 climbed sharply to a maximum of 2.39 g L-1 at 12 h, began to drop immediately after this high point and decreased to half of the maximum level by 33 h. This indicates that there was no stable citramalate production period without any additional plasmid stabilisation.

When the cer fragment was added to the plasmid (pQR2402), peak citramalate production was detected at the same time point, 12 h, and at a lower amount, 1.92 g L^-−1^. However, production was retained around maximum levels for 58 h before a drop in production was detected, reaching half this maximal level at 74 h. Total citramalate production can be inferred from the area underneath the concentration plot (Table 1). Despite the initially higher citramalate production, the increased stability provided by the cer fragment resulted in nearly 1.6 times the amount of chemicals production over the fermentation lifetime.

**Table 1:** The total citramalate production determined from the area underneath the concentration curve over the course of the 106 h continuous fermentation

Strain	Total citramalate production (g)	Bulk fluorescence detected (AU)
pQR2401	7.49	418555
pQR2402	12.22	767320
Ratio	1.58	1.83

Due to the operonic nature of the plasmid assembly, fluorescence is initially linked to expression of the citramalate gene. As a result, monitoring the fluorescence signal from samples taken during the fermentation allows plasmid expression and retention to be tracked by simple excitation/emission studies, simple spectroscopic analyses that are quicker, cheaper, and easier to scale than HPLC [20, 25]. Fluorescence readings can be performed in seconds, on pure samples, without the need for processing steps. Fluorescence values of samples from cells containing pQR2401 and cells containing pQR2402 both peaked at 20 h with cells containing pQR2401 dropping to half maximal fluorescence at 41 h and cells containing pQR2402 by 77 h. The delay compared to citramalate production may reflect maturation time delays required for fluorescence proteins [14]. Maturation time and stability can vary and the manufacturer does not provide this information, however in general, maturation for red fluorescent proteins has been shown to vary between approximately 0.5 h and 3 h [4]. Lysed cells may also release fluorescent proteins into the media which may be retained and thus give an extended reading for retention that does not accurately match productivity time. RFP is a useful indication of absolute gene retention and pathway expression levels, however, when measured as a bulk value it does not give a complete or accurate picture of how expression of pathway genes is affected over time. Spectroscopic analysis can, however, provide a more comprehensive understanding of phenomena that cannot be identified with conventional tools.

### Single-cell measurements of plasmid retention

The use of flow cytometry allows for single-cell level analysis giving more information than is available from bulk fluorescence or chemical concentration values alone. The ability to measure fluorescence levels of individual cells enables tracking of single-cell gene expression. The measurement of any fluorescent signal within a cell above a control value demonstrates retention of pathway expression (therefore no total plasmid loss and no global operon structural changes) and the value of the fluorescence of each cell demonstrates the expression levels of the operon at a single cell level.

When no additional stabilisation is present on the production plasmid, the percentage of cells retaining an RFP signal above control begins dropping after only 12 h (Fig. 4a, blue line), matching the observed citramalate production more closely than bulk fluorescence values. Cells seem to retain plasmid and expression during the batch phase, but non-expressing cells are accumulated as soon as the fermentation enters a carbon-limiting state. Addition of cer to the plasmid prevents accumulation of non-expressing variants arising until after 58 h (Fig. 4a, red line). This matches the profile of citramalate production showing that the single-cell fluorescence analysis is an excellent proxy for retention of plasmid-borne production genes. Half of the population no longer demonstrates RFP fluorescence by 45 h without stabilisation, increasing to 71 h when cer is included on the expression plasmid (Table 2).

**Table 2:** The calculated half-lives based on a regression fit of the RFP + percentage retention data, the time at which half of the cells no longer exhibit any fluorescence expression and are therefore likely to have lost plasmid, in a continuous fermentation at a dilution rate of 0.1 h^−1^

	pQR2401 (cer −)	pQR2402(cer +)
Half life (hours)	44.632	70.332
Overall productivity (g.L^−1^.h^−1^)	0.073	0.12
Peak productivity (g.L^−1^.h^−1^)	0.24	0.19
Product yield (g_citramalate_.g_glucose_^−1^)	0.13	0.21

The overall productivity across the entire fermentation and the peak productivity at the maximal citramalate productive period are shown as grams of product produced, per litre of fermentation broth, per hour. The product yield is shown as the amount of citramalate produced compared to the glucose added throughout the length of the fermentation

The median expression level of cells that retain an RFP signal above control increases throughout the continuous fermentation of pQR2401 containing cells, showing an increase of approximately four-fold from the steady state levels at 20 h to the maximum at 90 h (Fig. 4b). Although the proportion of cells retaining fluorescent protein expression is decreasing, those that still contain plasmids have an increased total expression level. In addition, the variance of this population increases in a similar way showing that the expression levels become variable as the fermentation progresses (Fig. 4c). It is likely that plasmids are being unevenly segregated at cell division, leading to a wide array of different copy numbers in the population, leading to a wide variety of pathway expression. In contrast, addition of the cer fragment to the expression plasmid results in a comparatively stable median fluorescence that peaks two-fold higher at 82 h than at 20 h, which demonstrates that cer has increased the likelihood of even plasmid segregation at cell division. Variation is lower throughout when cer is included in the expression plasmid, showing that expression levels are more consistent across the population. Median fluorescence does fall sharply when cer is present after 82 h, showing that pathway expression is still lost. It is possible that by this point in the fermentation structural instability may have become limiting.

The eventual loss of fluorescence in cer-containing cells suggests that cer does not guarantee high copy-number maintenance indefinitely but that it does decrease the likelihood of plasmid loss at each cell division. Eventually, loss of productivity does still occur, and non-productive plasmid free cells do arise. Further studies, potentially utilising next generation genomics techniques, will be needed to discover what is causing the loss of productivity when segregational stability has been increased with cer addition.

**Fig. 3:**
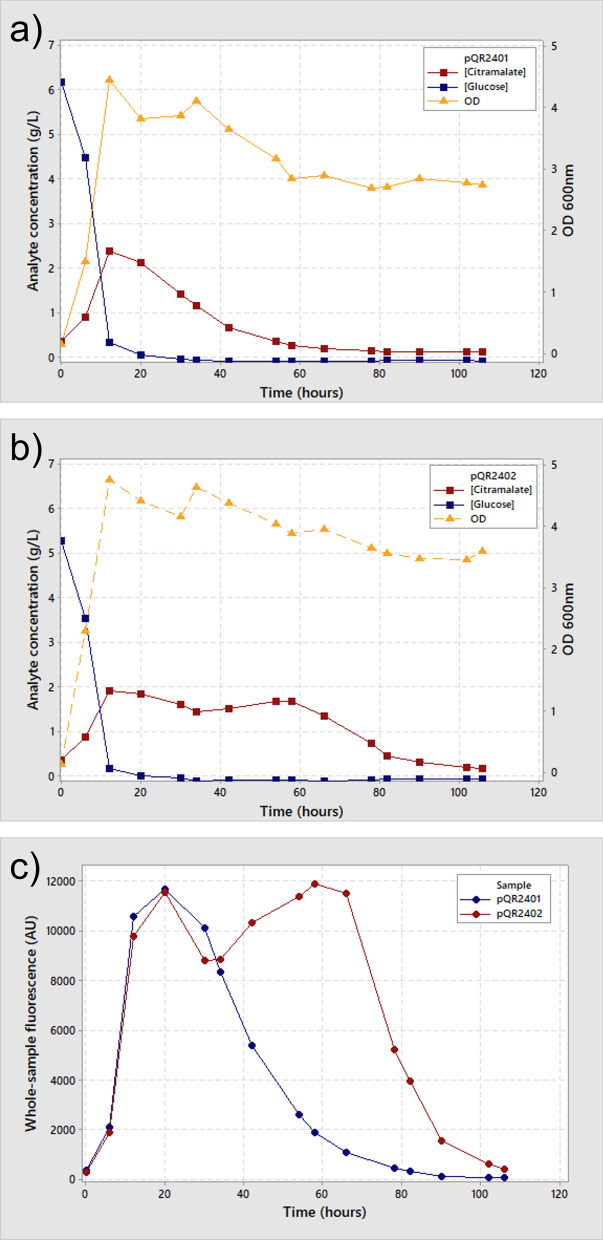
HPLC data showing the concentration of citramalate and glucose (primary axis), and OD_600nm_ (Secondary axis) for cells containing either plasmid pQR2401 (**a**) or pQR2402 (**b**). Graphs show glucose concentration, in blue, squares; citramalate concentration, in red, squares; and OD in orange, triangles. Samples were taken at intervals throughout a single continuous fermentation and concentration of analyte was determined by HPLC. A plot of bulk fluorescence values (**c**) shows the fluorescence signal from FresnoRFP for samples containing cells with either plasmid pQR2401 (blue circles) or plasmid pQR2402 (red circles) from the corresponding fermentation, determined from the excitation/emission (545/592) in a plate reader spectrometer

Peak productivity (i.e. the productivity when maximal citramalate production was achieved) was higher for cells lacking cer on the production plasmid, supported by the higher median fluorescence of cer- cells at this time. Inclusion of cer appears to stabilise the plasmid copy number, allowing for greater stability with a small reduction in maximial productivity. The overall productivity, however, was 60% higher when cer was included in the plasmid (Table 2). This reflects the trend seen in the concentration profiles (Fig. 3a, b) and demonstrates that without the addition of cer, cells lose productivity from their plasmids earlier in the continuous fermentation. This had a similar affect on the yield of the process, with cer + cells demonstrating 8% higher conversion of glucose to the citramalate product.

## Discussion

**Fig. 4:**
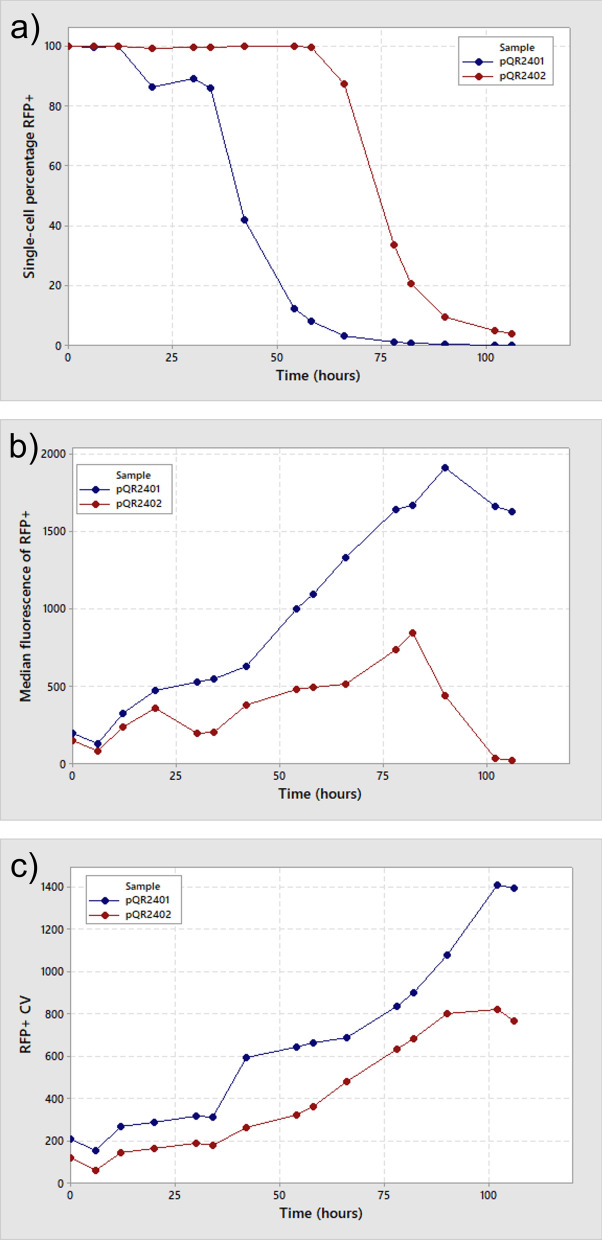
Single-cell fluorescence data from cells detected as having a fluorescence emission value above non-fluorescent control, and thus retaining fluorescent protein expression from the production operon, throughout the 106 h continuous fermentation. **A** The percentage of cells exhibiting a fluorescence reading above negative control and thus the proportion that still retain fluorescent protein expression from the production operon. **B** The median fluorescence value of cells that retain a fluorescent signal above control, indicating the comparative, average amount of fluorescent protein molecules in individual cells. **C** The coefficient of variance of this median fluorescence value and thus the variation in amounts of fluorescence protein molecules in cells across this population sample

Inclusion of cer on the expression vector in an *E. coli* host was previously shown to stably produce relevant production enzymes for 120 h, however, this was in a fed-batch-like process and the molecular basis for this stability or its subsequent loss was not able to be analysed further [11]. Here we show stable maximal production of an industrially relevant product, citramalate, for around 60 h in a continuous fermentation, merely by the inclusion of the cer site on the production plasmid and use single-cell analysis tools to elucidate molecular basis for this stability. We demonstrate productivity times 3.3 × higher with the inclusion of cer compared with a non-cer containing plasmid (Fig. 4). Increased stability generated by cer inclusion causes total production to be increased by more than 50% (Table 1). Previous studies have shown CimA3.7 expressing cells can obtain productivity over a 65 h fed-batch fermentation of 1.85 g.L^−1^.h^−1^ [33]. At a low dilution rate of 0.1 h^−1^, in a true continuous process, we demonstrate productivity of around 0.2 g.L^−1^.h^−1^. The higher cell densities generated previously are likely to enable very efficient conversion, however, in an industrial process, the need to generate product for longer periods is likely to be necessary to a cost-effective process. It is apparent that careful tuning of the dilution rate and substrate concentration is required to minimise substrate loss to cell growth. In addition, our use of constitutive promotors prevents toxic overexpression of plasmid components, which may mean that enzyme concentration is a limiting factor to conversion (albeit a factor that would be lessened also by increasing cell density).

The presence of a fluorescence marker linked to pathway expression here demonstrates excellent use as a proxy for productivity as well as retention. In addition, if used in conjunction with single-cell analyses it is possible to infer reasons for subsequent plasmid loss. Flow cytometry can determine single-cell fluorescence, generating more accurate data than bulk fluorescent measurements alone and correct gating of particles is able to limit any signal from lysed cells, ensuring that any signal relates more closely to transcribing, productive cells.

## Conclusions

Here we demonstrated the ability of single-cell fluorescence to determine operon retention and expression levels separately. This allowed the measurement of the proportion of cells retaining plasmid, and importantly, the proportion of cells retaining a functional, expressing plasmid. As a result, we were able to show that plasmids containing a cer multimer resolution site demonstrated increased retention, likely through decreasing the chance of plasmid free segregants arising. As plasmid-free segregants begin to arise in the strain containing the production plasmid lacking a cer multimer resolution site, the median fluorescence of those that retain plasmid increases. This suggests that plasmids are being unequally divided amongst the daughter cells. Some cells are inheriting a proportion of plasmid copies and are thus expressing more fluorescent protein (and thus production pathway proteins). However, the metabolic burden of these plasmid levels means these cells grow slower and are quickly outcompeted in the fermentation. Addition of cer to the production plasmid maintains a median expression level which suggests that daughter cells are inheriting roughly equal plasmid copy numbers. It is important to determine precisely how these cells still lose plasmid expression, as this will be the next step in prolonging productivity for industrially relevant timescales [13].

Once antibiotic selective pressure is absent, the only selective pressures that remain are against high plasmid copy number, due to the burden of the production pathway. Citramalate is produced from pyruvate in central metabolism, thus is competing directly with cell growth under glucose-limited conditions [2, 33, 35]. Inclusion of a cer site increases the chance of absolute plasmid retention but has no effect on selective pressure. The chance of a plasmid-free variant arising is decreased, but there is no selective drive to maintain plasmid, so once they arise, the fermentation is quickly taken over by non-productive variants. We show here that median fluorescence of cer-stabilised cells remains roughly constant until plasmid-free cells begin to arise (Fig. 4). It is only the Coefficient of Variance (CV) that rises gradually throughout, suggesting that variation and lack of selective pressure eventually gives rise to plasmid-free variants which are then able to rapidly take over the fermentation reaction. Generating a selective pressure towards plasmid retention (e.g. via an addiction system) should help to mitigate this loss further. This is the first report of how cer helps plasmid retention in continuous cultures and what the profile of loss looks like.

Statistically accurate comparison of fermentation data is difficult due to the media and equipment requirements of parallel studies. The ability to analyse multiple factors simultaneously would allow statistically accurate knowledge of the likelihood of segregants arising and speed of fermentation take-over once they do. The use of higher-throughput growth screens, coupled with the single-cell analyses used here, would allow the direct comparison of different stabilisation systems with greater statistical accuracy.

As discussed, addition of selective pressure elements (such as toxin/anti-toxin or metabolic addiction systems), also linked to pathway expression should increase plasmid stability further, however, structural changes are inevitably the limiting factor as is ultimately the case for cer stabilisation shown here. The next steps towards prolonging industrial-scale production times from bacteria should be focussed towards limiting mutations in the host and ensuring a pressure towards a higher copy number. Here we demonstrate the first step towards leveraging the power of synthetic biology tools to generate stabilised production strains for continuous fermentation. By streamlining these processes and analysing the further limiting factors to pathway gene retention, we are confident in producing stabilised strains to enable continuous processes for industrial bio-based production.

## Methods

### Strain construction

A vector backbone was generated from a pET29a( + ) (Novagen) plasmid by PCR to remove the Multiple Cloning Site (MCS) and expression cassette. A strong constitutive promoter (BBa_J23119) was selected from the Anderson Series of promotors and amplified along with the rfp gene, cimA3.7 gene and bidirectional terminator rrnB1 T1T2 (BioBrick promotors by JC Anderson) [19] (ATUM Bio). These were given complementary type IIS overhangs and a BsaI site to assemble into a two-gene operon using the established protocol [10]. Assemblies were transformed into NEB5a cells using standard methods (New England Biolabs) [26]. An identical backbone was used with an altered Type IIS site to provide an additional insert site for the cer fragment upstream of the rfp/cimA3.7 operon. Positive colonies were identified with kanamycin screening on Luria–Bertani (LB) Agar (1.2%), plasmid purified with NEB Monarch (New England Biolabs) and sequenced (Eurofins Scientific). Correct plasmids were transformed into electrocompetent BW25113 ΔldhA (cells using standard techniques [3, 26].

### Continuous fermentation

Continuous fermentation was performed in the Applikon my-Control system. General configuration for the fermenters was a total volume of 1.2 L, 2 Rushton impellers (separated by 4.5 cm), two baffles. Each bioreactor contained a working volume of 900 mL with a dilution rate (D) of 0.1 h^−1^. General operating conditions were at 37 °C, pH 7, 400 rpm and, a controlled dissolved oxygen (DO) at 30% saturation, cascade control for DO was activated and controlled by increasing air flow and if not sufficient, then increasing agitation rate up to a limit of 1400 rpm. Initial optical density (OD) was set at 0.1. Feeding and harvest were controlled by their respective pumps (part of the Applikon system) at 1.5 mL min^−^1 (D = 0.1 h^−1^). Polypropylene glycol (Sigma–Aldrich, MO) was added as antifoam at a ratio of 0.5 mL per L of fermentation volume. Sterile antifoam was added until accumulation of foam was observed to delay any possible influence on data collection. Total duration of the continuous fermentation was 106 h.

Cells were grown overnight on solid LB agar plate with Kanamycin grown 37 °C. A single colony was selected to inoculate liquid LB with Kanamycin and grown for 12 h (OD > 4) at 37 °C and 250 rpm. Afterwards, MS media containing Kanamycin was inoculated in flasks to an initial OD = 0.1 and grown (37 °C and 250 rpm) for 6–7 h for a final OD > 0.5. MS inoculum growth is affected greatly by media pH and subsequent decrease (data not shown), for this reason an additional phosphate buffer was included in this stage. Fermenters were inoculated at an OD = 0.1 and grown as explained before. Feed and harvest started at 6 h after inoculation.

Media composition is as follows: LB media consisted of tryptone (10 g L^−1^), yeast extract (5 g L^−1^), and NaCl (5 g L^−1^). MS media is composed of three individual parts and mixed after autoclave sterilization: KH2PO4 (2 g L^−1^) and trace element solutions (2 mL L^−1^); NH4Cl (for fermentation 4 g L^−1^ and for inoculum 3 g L^−1^) and MgSO4·7H2O (0.4 g L^−1^); 3) Glucose (5 g L^−1^) [15]. Trace element solution consists of EDTA (50 g L^−1^), ZnSO4 (2.2 g L^−1^—reduced from original recipe), CaCl2 (5.54 g L^−1^), MnCl2·4H2O (5.06 g L^−1^), FeSO4·7H2O (g L^−1^), (NH4)6Mo7O24·4H2O (1.1 g L^−1^), CuSO4·5H2O (1.57 g L^−1^), and CoCl2·6H2O (1.61 g L^−1^) [15, 31]. Media for continuous fermentation feeding had the same composition of MS media. For growth of inoculum in MS media, pH was controlled by potassium phosphate buffer 40 mM, pH 6. Final Kanamycin concentration was at 50 µg mL^−1^. All chemicals were obtained from Sigma–Aldrich (MO, USA) and NH4Cl from Merck (Germany).

### Plasmid expression analysis

100 µl of each time point was removed from each fermenter and analysed in a Clariostar microplate reader (BMG LABTECH) for absorbance at 600 nm and excitation/emission fluorescence at 545/592.

### HPLC

1 ml was also taken at each time point, spun at 17,000 × g for 1 min and the supernatant removed by pipetting. This was then filtered with a 0.22 µm filter and analysed with HPLC using an Aminex column with a mobile phase composed of TFA 0.1% v/v and a flowrate of 0.6 mL min^−1^.

### Flow cytometry

10 µl of each sample was taken and diluted in 1 ml 1 × Phosphate Buffered Saline without Calcium and Magnesium (Lonza). Single-cell fluorescence was analysed on a BD FACS Jazz with 561 nm laser excitation and emission detection with a 585/29 nm filter. 100,000 particles were read for each sample. Particles were separated into gates initially to remove non-cell matter from a blank media control. Control cells without fluorescent plasmid were then used to determine gate that detected 99% of non-fluorescent cells. RFP + gate was then taken as any fluorescent reading exceeding this control gate. A positive control consisting of cells containing plasmid and grown overnight in LB media at 37 °C with antibiotic selection was used to ensure RFP + gate returned 99% of particles demonstrating RFP expression.

## Supplementary Information

**Additional file 1: Table S1.** The sequence appended to DNA parts and their corresponding ID. The sequences were appended to primers used to generate part PCR products to generate specific overhangs when digested with BsaI in a type IIS restriction/ligation reaction. Black text sequences show either a 6bp extra length of DNA to ensure efficient BsaI binding, or the single spacer nucleotide between the recognition site and the generated overhang. Blue sequence text represents the BsaI recognition site and red sequence text represents the sequence of the overhang generated after restriction digestion with BsaI.

## Data Availability

The datasets used and/or analysed during the current study are available from the corresponding author on reasonable request.
