# Supplementary material for: Segregationally stabilised plasmids improve production of commodity chemicals in glucose-limited continuous fermentation
Source: Microb Cell Fact. 2022 Nov 3;21:229. doi: 10.1186/s12934-022-01958-3 (PMC9632041; doi:10.1186/s12934-022-01958-3)
Supplement: Supplementary file 1 — Additional file 1: Table S1. The sequence appended to DNA parts and their corresponding ID. The sequences were appended to primers used to generate part PCR products to generate specific overhangs when digested with BsaI in a type IIS restriction/ligation reaction. Black text sequences show either a 6bp extra length of DNA to ensure efficient BsaI binding, or the single spacer nucleotide between the recognition site and the generated overhang. Blue sequence text represents the BsaI recognition site and red sequence text represents the sequence of the overhang generated after restriction digestion with BsaI. [file 12934_2022_1958_MOESM1_ESM.docx]

# Supplementary Data

| Site ID | DNA sequence (6bp extra, BsaI cut site, overhang) |
| --- | --- |
| 1 | ACAACGGGTCTCTGGAG |
| 2 | ACAACGGGTCTCTAATG |
| i | ACAACGGGTCTCTTACT |
| ii | ACAACGGGTCTCTAATG |
| iii | ACAACGGGTCTCTAGGT |

Table S1 The sequence appended to DNA parts and their corresponding ID. The sequences were appended to primers used to generate part PCR products to generate specific overhangs when digested with BsaI in a type IIS restriction/ligation reaction. Black text sequences show either a 6bp extra length of DNA to ensure efficient BsaI binding, or the single spacer nucleotide between the recognition site and the generated overhang. Blue sequence text represents the BsaI recognition site and red sequence text represents the sequence of the overhang generated after restriction digestion with BsaI.
